# High Fragmentation Characterizes Tumour-Derived Circulating DNA

**DOI:** 10.1371/journal.pone.0023418

**Published:** 2011-09-06

**Authors:** Florent Mouliere, Bruno Robert, Erika Arnau Peyrotte, Maguy Del Rio, Marc Ychou, Franck Molina, Celine Gongora, Alain R. Thierry

**Affiliations:** 1 SysDiag UMR3145 – CNRS, National Centre of the Scientific Research/BIO-RAD, Montpellier, France; 2 U896 INSERM, National Institute of Health and Medical Research, University Montpellier1, IRCM, Institute of Oncological Research of Montpellier, Montpellier, France; 3 CRLC, Regional Centre against Cancer, Val d'Aurelle-Paul Lamarque, Montpellier, France; University of Hong Kong, Hong Kong

## Abstract

**Background:**

Circulating DNA (ctDNA) is acknowledged as a potential diagnostic tool for various cancers including colorectal cancer, especially when considering the detection of mutations. Certainly due to lack of normalization of the experimental conditions, previous reports present many discrepancies and contradictory data on the analysis of the concentration of total ctDNA and on the proportion of tumour-derived ctDNA fragments.

**Methodology:**

In order to rigorously analyse ctDNA, we thoroughly investigated ctDNA size distribution. We used a highly specific Q-PCR assay and athymic nude mice xenografted with SW620 or HT29 human colon cancer cells, and we correlated our results by examining plasma from metastatic CRC patients.

**Conclusion/Significance:**

Fragmentation and concentration of tumour-derived ctDNA is positively correlated with tumour weight. CtDNA quantification by Q-PCR depends on the amplified target length and is optimal for 60–100 bp fragments. Q-PCR analysis of plasma samples from xenografted mice and cancer patients showed that tumour-derived ctDNA exhibits a specific amount profile based on ctDNA size and significant higher ctDNA fragmentation. Metastatic colorectal patients (n = 12) showed nearly 5-fold higher mean ctDNA fragmentation than healthy individuals (n = 16).

## Introduction

Recent advances in the understanding of the molecular mechanisms of cancer enlarge the scope of diagnostic strategies in oncology [1]. Many genetic alterations involved in tumourigenesis are now positioned within a time sequence [2]. Moreover it is fully acknowledged that tumours release cell-free nucleic acids in biological fluids, especially blood [3]. Therefore, the detection of the genetic alterations associated with tumour development in circulating DNA (ctDNA) appears to be a particularly attractive approach as a non-invasive, diagnostic or theranostic test for cancers [4]. Previously, ctDNA was used as biomarker in two ways: first by measuring its concentration and second by studying its nucleotidic sequence [4], [5]. Moreover, ctDNA levels in plasma have been shown (i) to be significantly higher in CRC patients than in healthy individuals [6]; (ii) to decrease progressively in the follow-up period in tumour-free patients; and (iii) to increase in patients with cancer recurrence or metastasis [5], [7], [8]. Many studies have been focusing on the identification of abnormal forms of DNA in plasma or serum; however, the reported results are so far contradictory, although very high rates of cancer detection have been obtained in such a way. These reports, although promising, have led to many questions about the reliability of using abnormal ctDNA as cancer biomarker [9], [10]. Particularly, it is imperative to develop technologies that can detect the number of mutant DNA molecules and the specific mutation(s) in the same sample. For this it is necessary to improve the sensitivity and specificity of ctDNA analysis. In addition, in order to be able to compare results across samples and studies, it is necessary to have detailed information about the amount and the size distribution of ctDNA fragments and to compare the data obtained for neoplastic and non-neoplastic cell ctDNA. Necrosis, which is associated with tumour development, as well as apoptosis and phagocytosis, which are associated with defence mechanisms, lead to destruction of tumour cells and also of the adjacent, non-tumour tissues [11], [12], [13], [14], and to DNA fragmentation as an hallmark of apoptosis. However, fragmentation of cell-free DNA is higher following apoptosis than following necrosis or phagocytosis [13]. Specifically, ctDNA fragments longer than 10,000 bp are likely to originate from necrotic cells, whereas DNA fragments shorter than 1000 bp, particularly of 180 bp or multiples of this size, are reminiscent of the oligonucleosomal DNA ladder observed in apoptotic cells [15]. Thus, a detailed analysis of the size of the ctDNA fragments could allow discerning the source of circulating nucleic acid in cancer patients. To this aim we developed a highly specific Q-PCR assay that has already allowed us to unequivocally identify tumour and non-tumour derived ctDNA released during cancer progression in an animal model (athymic nude mice xenografted with human colon cancer cells) [16]. Here, we have used this assay to evaluate the amount and size distribution of non-tumour and tumour-derived ctDNA fragments. We then have applied the same system to quantify ctDNA fragments and their size distribution in plasma samples from healthy individuals and patients with metastatic colorectal cancer (CRC). Our data revealed that analysis of ctDNA fragmentation provides valuable information relative to cancer diagnosis.

## Materials and Methods

### 1. Cell lines

Human HCT116-s [17], SW620 (ATCC: CCL-227) and HT29 (ATCC: HTB-38) cells and mouse MC38 [18] colon carcinoma cell lines were maintained in RPMI + 10% fetal bovine serum.

### 2. Xenograft model

Female athymic nude mice (6–8 wk/old) were purchased from Harlan (Gannat, France) and maintained in a specific pathogen-free facility (study approval N° B-34-172-27; Institut de Recherche en Cancérologie de Montpellier-CRLC Val d'Aurelle-Paul Lamarque, Montpellier, France). Mice were xenografted subcutaneously with 2×10^6^ cancer cells. Mice were euthanized with CO2 at different time points post-graft according to the tumour weight desired. Peripheral blood was drawn into EDTA pre-coated tubes and was used for plasma preparation within one hour. All experiments complied with the current national and institutional regulations and ethical guidelines and were performed by an accredited person (Dr. B. ROBERT, N°34-156).

### 3. Human blood samples

Blood samples from patients with metastatic colorectal cancer (CRC) (n = 12) and from selected human healthy individuals (human healthy plasma, HHP, n = 16) were collected in EDTA tubes. The overall process from blood collection to plasma preparation did not exceed 3 hours and plasma was stored at −80C°. Written, informed consent was obtained from all participants prior to the onset of the study. Protocols for the use of blood of healthy volunteers used in this study were approved by the “Etablissement Français du Sang” (EFS) Ethics Committee (EFS-PM agreement: 21/PVNT/MTP/CNR14/2010-0029). According to the Code de Santé Publique Article L1131-1 and next, no specific ethical approval is required for this type of study. Each CRC and HHP sample was numbered (CRCn and HHPn) and corresponded to the plasma of a single subject with the exception of HHP3, which was a pool of 6 different plasma samples. TNM classification of CRC 1–4 patients correspond to T4N3M1, T3N2M1, T4N1M1 and TxN2M1, respectively. All metastatic CRC patients did not received chemotherapy at least one month before blood collection.

### 4. Plasma preparation

Mouse and human blood was collected in 4 ml Venosafe K2E tubes (Terumo) and centrifuged at 1200 g at 4°C in a Heraeus Multifuge LR centrifuge with a CR 4j rotor for 10 minutes. Supernatants were isolated in sterile 1.5 ml Eppendorf tubes and centrifuged at 16 000 g at 4°C for 10 min. Then, supernatants were either immediately handled for DNA extraction or stored at −80°C. No significant difference was found in Q-PCR assays when comparing freshly isolated or stored plasma.

### 5. DNA extraction

ctDNA was isolated from 200 µl plasma with the QIAmp DNA Mini Blood kit (Qiagen, CA) according to the “Blood and body fluid protocol” in an elution volume of 60 µl. DNA samples were kept at −20°C until use. No significant difference was found in the results of the Q-PCR assays in which freshly extracted or stored DNA was used.

### 6. Oligonucleotides

The oligonucleotide primers used in this work are described in Table S1. Primers were designed and selected according to the previously described methods [16].

### 7. CtDNA quantification by Q-PCR

The Q-PCR method and ctDNA analysis were previously described in detail [16]. Methodology and data description were carried out according to the MIQE guidelines [19]. Q-PCR amplifications were carried out in a reaction volume of 25 µl on a Chromo4 instrument using the MJ Opticon Monitor 3 software (Bio-Rad). Each PCR reaction mixture was composed of 12.5 µl PCR mix (Bio-Rad Super mix SYBR Green); 2.5 µl of each amplification primer (0.3 pmol/µl); 2.5 µl PCR-analyzed water and 5 µl DNA extract. Thermal cycling was organised in 3 repeated steps: a first denaturation step of 3 min at 95°C, followed by 40 repeated cycles of 95°C for 10 sec and 60°C for 30 sec. Melting curves were obtained by increasing the temperature from 55°C to 90°C with a plate reading every 0.2°C., Serial dilutions of genomic DNA from human placenta cells were used as calibrators for quantification, their concentration being assessed with a Nanodrop spectrophotometer. Each sample was analyzed in duplicate and each assay repeated at least once. The ctDNA concentrations obtained with each primer set were normalized to the precise concentration of a genomic DNA sample amplified using the same primer set. The coefficient of variation due to ctDNA extraction and of concentration value was calculated as 4% and 19% respectively (n = 12). The ctDNA amount was arbitrarily estimated for the 60–100, 100–150, 150–400 and >400 bp fragment size ranges. ctDNA amount was determined for 1 ml and expressed as ng. The estimated amount of ctDNA in each range of ctDNA size was calculated by subtracting the amount obtained with the larger amplicon from the amount obtained with the shorter amplicon (100 bp minus 60 bp, 150 bp minus 100 bp, 400 bp minus 150 bp). The fraction corresponding to ctDNA fragment higher than 400 bp corresponds to the amount obtained when targeting sequence of 382 bp (mouse) or 409 bp (human).

### 8. Estimation of ctDNA fragmentation

CtDNA fragmentation was assessed by calculating an index we termed DNA Integrity Index (DII). DII was calculated as the ratio of larger/shorter fragment concentrations. DII is therefore theoretically 1 if the template DNA is not truncated and <1 if it is truncated into fragments smaller than the template. Conventionally, long and short fragments were longer or shorter than 180 bp (i.e., the mononucleosome average size). To note, DII did not significantly vary when Q-PCR analysis was either carried out 2 hours following blood sampling or after plasma freezing, provided that no blood degradation was observed after 4 hours in EDTA (ethylenediaminetetraacetic acid) tubes at room temperature (data not shown). In addition, samples were handled in the same way in the same experiments.

### 9. Q-PCR targeted sequences

**Figure 1:**
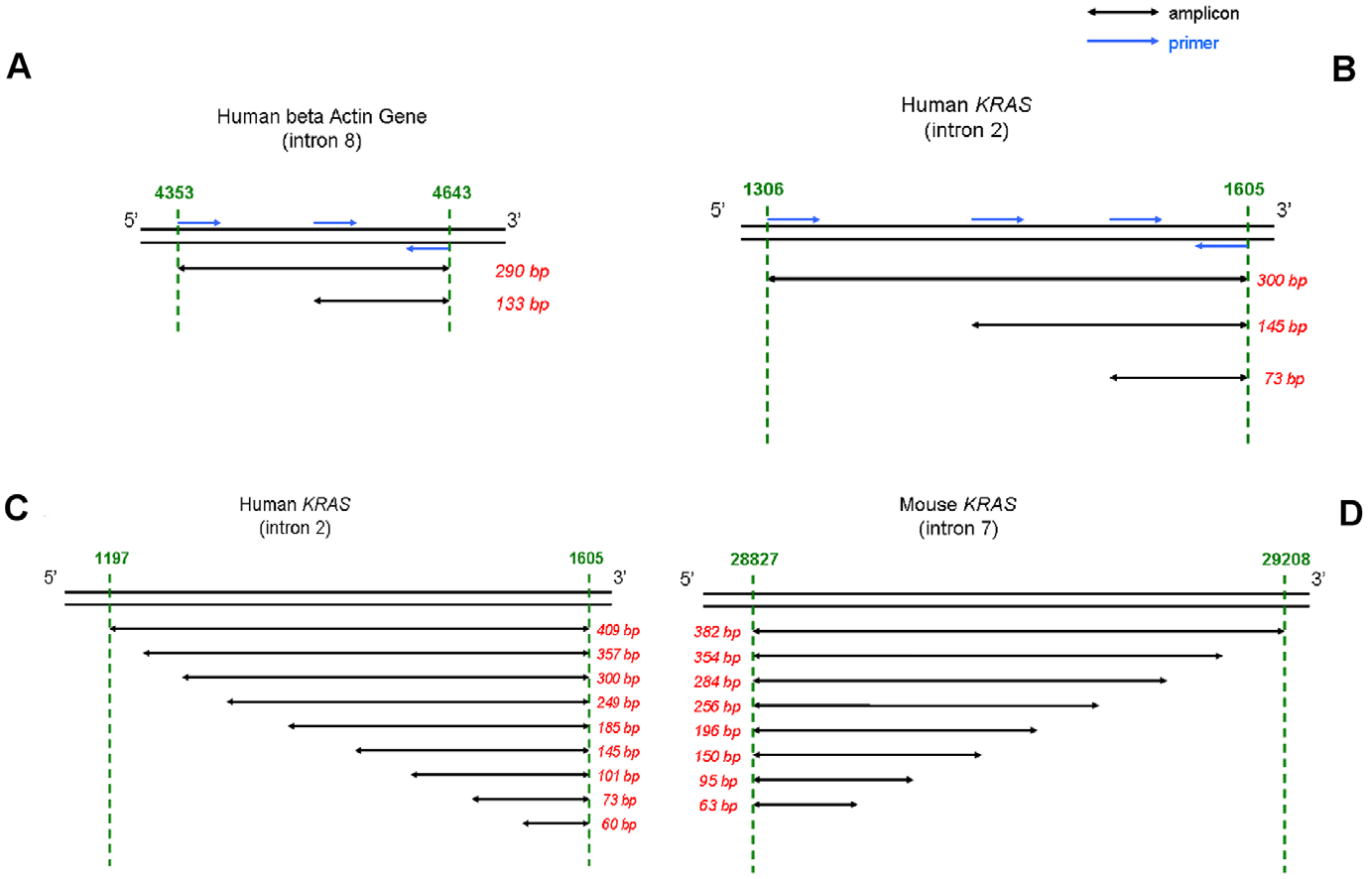
Primer designs used in the study. Primer targeting an *ACTB* region for quantifying ctDNA and determining ctDNA fragmentation from HT29 xenografted mice (**A**). Primer targeting a human *KRAS* region for studying the effect of the target sequence size on ctDNA concentration and fragmentation in plasma from SW620 xenografted mice (**B**). Primer design for studying the size distribution profile of ctDNA fragments in plasma from SW620 xenografted mice and human individuals by targeting human (**C**) and mouse (**D**) *KRAS* region.

Targeted sequences are located in two genes: *KRAS* and *ACTB (* Table S1 *)*. Evaluation of ctDNA fragmentation was performed using an original double integrated PCR system that targets human *ACTB* sequences [16] or intron 2 of the mouse or human *KRAS* gene in which the reverse primer was the same for the short and the long fragment (Fig. 1, A and B).

The amount profile of ctDNA fragments of different size was assessed using an integrated PCR system that target intronic sequences of mouse or human origin within the same region. For the human wild type specific primer set the designed sizes of amplification were 60 bp, 73 bp, 101 bp, 145 bp, 185 bp, 249 bp, 300 bp, 357 bp and 409 bp (the reverse primer was the same for each amplification system). These primers targeted intron 2 of human *KRAS* (Fig. 1, C). For the mouse wild type specific primer set the designed sizes of amplification were 63 bp, 95 bp, 150 bp, 196 bp, 256 bp, 284 bp, 354 bp and 382 bp (the reverse primer was the same for each amplification system). These primers targeted intron 7 of mouse *KRAS* (Fig. 1, D). The median of the non-specific amplification of the mouse and human *KRAS* primer sets, assessed by determining the concentration profile of aliquots of human placental DNA and of MC38 DNA, was 7% and 9%, respectively.

### 10. Statistical analysis

Data were expressed as mean ± SD. The Student's *t*-test was used for comparison of means. A probability of less than 0.05 was considered to be statistically significant; *p ≤ 0.05, **p ≤ 0.01, ***p ≤ 0.001.

## Results

### 1. ctDNA fragmentation increases with tumour size and tumour ctDNA concentration

**Figure 2:**
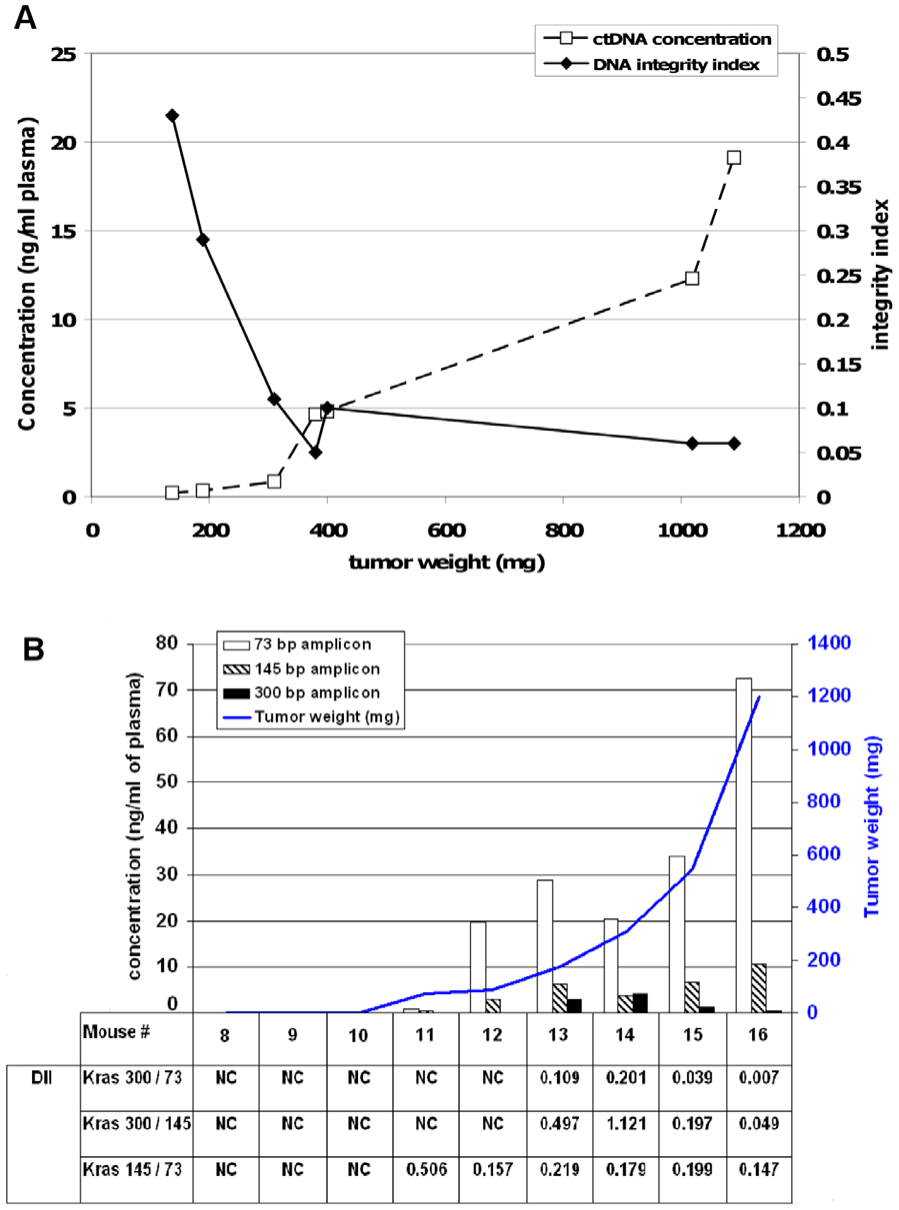
Evolution of tumour-derived ctDNA fragmentation and concentration relative to tumour weight. (**A**): Athymic nude mice were xenografted with HT29 cells and sacrificed at various time points and thus with tumours of increasing weight (137, 189, 310, 380, 400, 1019, 1080 mg from Mo1 to Mo7, respectively). CtDNA concentration (ng/ml plasma, left y axis) (dashed line) was assessed by Q-PCR using a primer set that amplifies a 133 bp sequence from human *ACTB* (Fig. 1A). Tumour-derived ctDNA fragmentation (full line) was estimated by calculating the DII (right y axis). Here, the DII corresponds to the ratio of the concentration found when targeting a 290 bp sequence of the human *ACTB* gene to the concentration found when targeting a 133 bp sequence located within the former 290 bp region (Fig. 1A). (**B**): Tumour ctDNA was quantified by Q-PCR with human *KRAS* 73 (empty bars), *KRAS* 145 (hatched bars) and *KRAS* 300 (full bars) primer sets (Fig. 1B) in plasma samples from SW620 xenografted mice. Mo8, Mo9 and Mo10 correspond to three non-xenografted nude mice. CtDNA fragmentation was estimated by calculating the 300/145 and 300/73 DII. DII was calculated as the ratio of the ctDNA concentrations obtained from the amplification of a short 73 bp, a medium 145 bp and a long 300 bp target sequence from the human *KRAS* gene (Fig. 1B). Bars represent ctDNA concentration expressed as ng/ml of plasma (left y axis) and curve represents tumour weight (mg, right y axis). Data represent the mean values of the ctDNA concentrations obtained in duplicates. NC, not calculated. No ctDNA was detected with the *KRAS* 300 bp primers in Mo11 and Mo12 plasmas.

Nude athymic mice xenografted with HT29 cells were sacrificed at different time points, tumours were weighted and ctDNA concentration in plasma samples determined using the HuACTB 133 primer pair that targets a 133 bp sequence in the human *ACTB* gene (Fig. 2, A). The concentration of ctDNA increased slightly (from 0.23 to 4.8 ng/ml) in samples from mice bearing small tumours (<500 mg), but then rapidly augmented up to 19 ng/ml in samples from mice with bigger tumours (>500 mg). Then, the corresponding DII was calculated using the ctDNA concentrations obtained with this primer pair and the values obtained with the HuACTB 290 primer set that amplifies a longer amplicon (290 bp). DII sharply decreased (from 0.44 to 0.1) in samples from mice with tumours up to 400 mg and then remained constant at approximately 0.05, notwithstanding the steadily increase in tumour weight.

### 2. ctDNA quantification by Q-PCR highly varies according to the amplicon length

Since the previous results indicate that ctDNA fragmentation increases with tumour progression, we then tested using our PCR system whether the evaluation of ctDNA concentration could be influenced by the amplicon length. To this aim, the concentration of tumour ctDNA in plasma samples of mice xenografted with SW620 cells was quantified using three human *KRAS* primer pairs that amplify sequences of 73 bp, 145 bp and 300 bp (Fig. 2, B) and with *KRAS* primers targeting mouse and human *KRAS* (214 and 189 bp, Figure S1). With the *KRAS* 300 primers (300 bp amplicon) ctDNA was not detected in plasma samples from the mice with the smallest tumours (<174 mg), but then, in samples from mice with tumours heavier than 174 mg, the ctDNA concentration decreased progressively with the increase of tumour weight. On the other hand, the ctDNA concentrations obtained using the *KRAS* 145 primer set seemed to slightly increase with tumour size. Conversely, with the *KRAS* 73 primers the ctDNA concentration highly increased in parallel with tumour weight. Data obtained by detecting 145 and 73 bp *KRAS* amplicon confirmed the increase of ctDNA concentration with tumour weight observed in HT29 cells by detecting 133 bp *ACTB* amplicon (Fig. 2, A). These results indicate that the quantification of ctDNA concentration is strongly influenced by the choice of the amplicon length. For instance, the ctDNA concentration in mouse #16 determined with the *KRAS* 73 primers was 5.3- and 40.5-fold higher than the concentrations obtained with the *KRAS* 145 and *KRAS* 300 primer pairs, respectively. The DII was then calculated (Fig. 2, B). Although both KRAS 300/145 and KRAS 300/73 DII decreased with tumour progression (13- and 30-fold decrease, respectively), KRAS 300/145 DII remained more elevated than 300/73 DII (for instance the mouse #16 DII was 0.13 for the KRAS 300/145 ratio and 0.02 for the KRAS 300/73 ratio).

### 3. Tumour-derived ctDNA fragments exhibit a specific size distribution in xenografted mice

**Figure 3:**
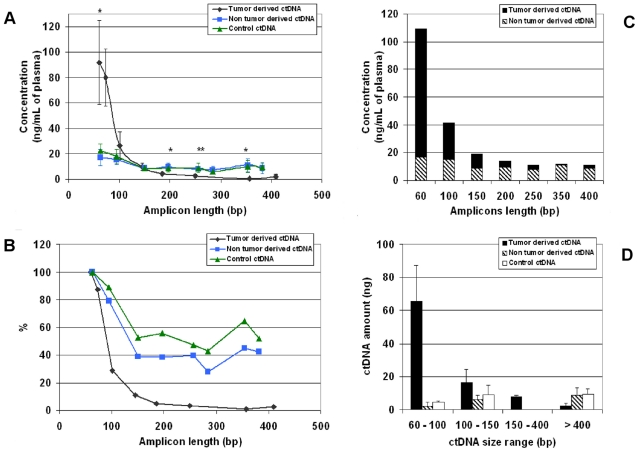
CtDNA fragment size profiles determined by Q-PCR for amplicons of different length in xenografted mice. Evaluation of tumour and non-tumour ctDNA concentration in plasma samples from mice xenografted with SW620 cells and of control ctDNA in non-xenografted nude mice was determined using a multi-integrated Q-PCR system for detecting amplicons of increasing length in intron 2 of human wild type *KRAS* (Fig. 1) or in intron 7 of mouse wild type *KRAS* (Fig. 1). Tumour ctDNA, non-tumour ctDNA and control ctDNA concentrations are expressed as ng/ml of plasma (**A**) or as % of the highest value of each concentration set (**B**). Each point represents the mean of three pools of plasma from three mice with 300–550 mg tumours. The relative percentage of tumour and non-tumour ctDNA from xenografted mice for each amplicon length is presented in (**C**): Tumour (black bars) and non-tumour (hatched bars) ctDNA mean concentrations are expressed as ng/ml of plasma; the bar height is the sum of tumour and non-tumour ctDNA concentrations (estimated as the total ctDNA concentration). For clarity human and mouse amplified targeted sequences of close size were grouped together: 60 (60 and 63), 100 (101 and 95), 150 (145 and 150), 200 (185 and 196), 250 (249 and 256), 350 (354 and 357) and 400 (409 and 382) bp. Fractional fragment size distribution of ctDNA amount from tumour and non-tumour ctDNA in xenografted mice and control ctDNA in non-xenografted mice (**D**). The ctDNA amount was arbitrarily estimated for the 60–100, 100–150, 150–400 and >400 bp fragment size ranges. The estimated amount of ctDNA (ng) in one ml of plasma in each range of ctDNA size was calculated as described in Materials and Methods section.

The previous results indicate that quantification of tumour ctDNA is influenced by the amplicon size. We thus asked whether the size distribution profiles of neoplastic and non-neoplastic ctDNA fragments were different. In order to reduce as much as possible the effect of tumour size, ctDNA was quantified by calculating the mean concentration values in three pools of plasma from three mice with tumours weighing between 300 and 550 mg (Fig. 3). The concentration profile of the different non-tumoral ctDNA fragments in these pooled samples, obtained using mouse *KRAS* primers that amplify fragments of increasing length up to 382 bp, was similar to that of control, non-xenografted mice (Fig. 3A and B). With human *KRAS* primers that amplify tumour-derived ctDNA fragments of increasing length up to 409 bp, the highest ctDNA concentrations were obtained for amplicons of length <100 bp and were much higher than the values obtained for mouse (non-tumoral and control) ctDNA. Conversely, when detecting amplicons of size ranging from 100 to 150 bp comparable levels of non-tumour, tumour-derived and control ctDNA were obtained, and for amplicons >185 bp lower concentrations of tumoral than non-tumoral ctDNA were found (p = 0.047 and 0.007 for the 185 bp and 250 bp amplicons, respectively). These results indicate that 1) ctDNA fragments smaller than 150 bp are predominantly detected in tumour ctDNA extract; 2) the proportion of ctDNA of tumour origin is inversely correlated with the amplicon size (from less than 20% of the total detectable ctDNA for the 400 bp amplicon to 85% for the 60 bp amplicon) (Fig. 3C). Finally, ctDNA amount upon arbitrarily selected size ranges (Fig. 3D) highlights that tumour-derived ctDNA amount could be easily discriminated versus non tumour and control ctDNA when targeting sequence <100 bp (65 vs 2–5 ng) and when targeting sequence >400 bp (2 vs 9–10 ng).

### 4. The specific size distribution profile of tumour ctDNA fragments appears to be relevant also in CRC patients

**Figure 4:**
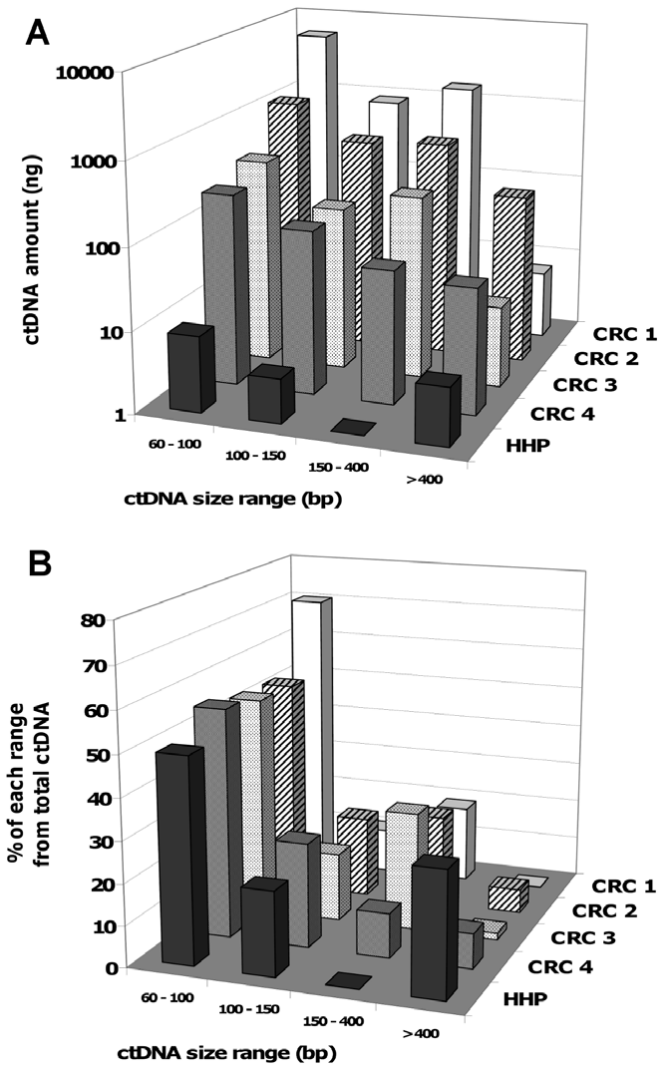
ctDNA size profile upon amplicon length. Comparison of ctDNA size profile upon amplicon length in healthy individuals (HHP) and CRC patients (**A**). CtDNA amount was calculated as described in Material and Methods section. The corresponding concentration values for each CRC and HHP plasma sample are presented in Table 1. HHP is the mean from plasmas of two healthy individuals (HHP1 and HHP2) and of a pool of 7 healthy individuals (HHP3). The estimated amount of ctDNA in each range of ctDNA size was calculated as described in Materials and Methods section. HHP and CRC ctDNA concentration are expressed as % of total ctDNA value of each profile (**B**). CtDNA concentration was determined using a multi-integrated Q-PCR system for detecting amplicons of increasing length in intron 2 of human wild type *KRAS* (Fig. 1) as performed in Fig. 3.

The same human *KRAS* primer sets that amplify DNA fragments of different size (Fig. 1, C) was used to study the ctDNA size distribution in plasma samples from healthy individuals (HPP) and four metastatic CRC patients who were not under treatment (CRC1 to CRC4) (Fig. 4). Overall higher ctDNA concentrations were detected in CRC plasma samples than in HHP samples (Table 1). For instance, the plasma DNA concentrations determined with the primers that amplify the 60 bp amplicon were 8078, 2134, 618 and 402 ng/ml for CRC1, CRC2, CRC3 and CRC4 and 24.8, 11.2 and 18.4 ng/ml in HHP1, HHP2 and HHP3, respectively. Moreover, whereas among CRC patients ctDNA levels were very different, in healthy individuals they did not vary much as the mean +/− SD was 18.1+/−6.8. To note, the value of HHP3, which is a pool of plasmas from 7 different healthy donors, was similar to that of the individual HHP1 and HHP2 samples. The mean ctDNA amount in human healthy individuals was found maximal at 60–100 bp (49%) range while being very low at 150–400 bp range (1%). Except for the >400 bp range HHP ctDNA amount was much lower (of several order) than that of CRC ctDNA amount observed when fragments are <400 bp. In particular, CRC ctDNA amount observed within 150–400 bp rise to 178–1520 ng whereas HHP amount was very poor (<1 ng). Conversely, the relative CRC ctDNA amount detected for fragment >400 bp appears very low (1–5%), suggesting that the relative determination of ctDNA amount within a fragment size range might distinguish healthy vs CRC plasmas. In this regard, the 300/60 DII calculated from the concentration values obtained when detecting target sequence of 300 and 60 bp, was 0.45 for the HHP and 0.055 for the CRC samples (Table 1).

**Table 1:** Total ctDNA concentration (ng/ml plasma) and DII in plasma samples from metastatic CRC patients (CRC1-4) and healthy individuals (HHP1-3).

Samples	Amplicons sizes (bp)								
	60	73	101	145	185	249	300	357	409
**CRC 1**	8077.8	6004.8	2395.5	1526.2	815.2	275.9	179.3	162.6	6.8
**CRC 2**	2133.9	2326.0	1016.5	596.2	409.3	267.2	175.1	105.1	123.9
**CRC 3**	618.1	591.5	289.9	187.7	93.2	46.1	37.5	20.3	9.9
**CRC 4**	402.2	286.3	179.0	77.4	81.3	135.3	62.9	28.1	34.1
**HHP 1**	24.8	16.5	9.8	5.9	7.0	6.0	3.6	2.4	3.0
**HHP 2**	11.2	12.8	7.6	6.1	6.4	6.8	8.0	5.2	7.4
**HHP 3**	18.4	17.3	11.6	6.4	5.2	9.8	9.0	4.4	4.7

Samples	Integrity index								
	60/60	73/60	101/60	145/60	185/60	249/60	300/60	357/60	409/60
**CRC 1**	1.000	0.743	0.297	0.189	0.101	0.034	0.022	0.020	0.001
**CRC 2**	1.000	1.090	0.476	0.279	0.192	0.125	0.082	0.049	0.058
**CRC 3**	1.000	0.957	0.469	0.304	0.151	0.075	0.061	0.033	0.016
**CRC 4**	1.000	0.712	0.445	0.192	0.202	0.336	0.156	0.070	0.085
**mean CRC**	**1.000**	**0.876**	**0.422**	**0.241**	**0.161**	**0.143**	**0.080**	**0.043**	**0.040**
**HHP 1**	1.000	0.664	0.394	0.239	0.284	0.244	0.147	0.098	0.123
**HHP 2**	1.000	1.145	0.680	0.547	0.571	0.607	0.712	0.463	0.659
**HHP 3**	1.000	0.941	0.628	0.348	0.282	0.531	0.490	0.237	0.257
**mean HHP**	**1.000**	**0.916**	**0.567**	**0.378**	**0.379**	**0.461**	**0.450**	**0.266**	**0.346**
**HHP/CRC**	**1.000**	**1.047**	**1.345**	**1.568**	**2.348**	**3.232**	**5.597**	**6.180**	**8.673**

DII was evaluated by calculating the ratio between the concentration of each amplicon and that of the 60 bp amplicon. HHP/CRC is the ratio between the mean HHP DII and the mean CRC DII.

### 5. Comparison of the fractional size distribution of ctDNA in CRC patients and xenografted animals

**Figure 5:**
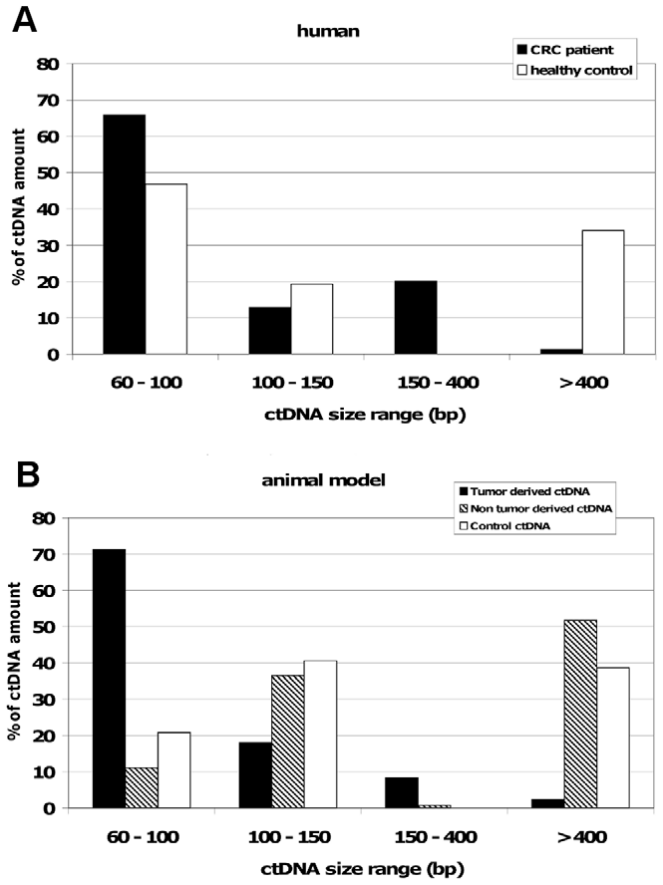
Comparison of the fractional fragment size distribution of ctDNA from clinical samples and from the animal model. CRC and healthy patients (**A**) and tumour and non-tumour ctDNA in xenografted mice and control ctDNA in non-xenografted mice (**B**). The estimated amount of each ctDNA fraction was expressed as the percentage to the total ctDNA amount estimated as the sum of the ctDNA amount of the four size fractions. Data were calculated from the experiments presented in Table 1, Fig. 3 (mouse plasma samples) and Fig. 4 (human plasma samples).

Figure 5 shows the relative ctDNA amount from plasmas of human (Fig. 5A) and mouse (Fig. 5B) origin. The percentage of 60–150 bp ctDNA was rather similar in HHP and CRC samples despite the higher proportion of tumour ctDNA observed for the 60–100 bp range. Decreased but significant ctDNA amounts for the 150–400 range were detected in CRC samples, but not in HHP samples. Conversely, the amount of >400 bp ctDNA in HHP samples (approximately 35%) was 17-fold higher than in CRC samples (2%).

A similar method of evaluation of the ctDNA amount was employed for the data (Fig. 3) obtained in xenografted mice (Fig. 5, B). The amount of mouse ctDNA relative to fragment size range was comparable in non-xenografted (control) and xenografted mice (non-tumoral) and its distribution profile was similar to the one observed in healthy controls (Fig. 5, A) with a noticeable very low percentage in the 150–400 size range and a significant higher proportion of >400 bp ctDNA (39% and 52% for control and non-tumoral mouse ctDNA). Conversely, the percentage of tumour-derived ctDNA in the 60–100 bp range was very high (71%) and decreased sharply to 2% for fragments >400 bp. The fractional fragment size distribution of tumour-derived ctDNA from xenografted mice and CRC patients was comparable (Fig. 5A and B).

### 6. Comparison of the DII in healthy and CRC patients and in xenografted animals

**Figure 6:**
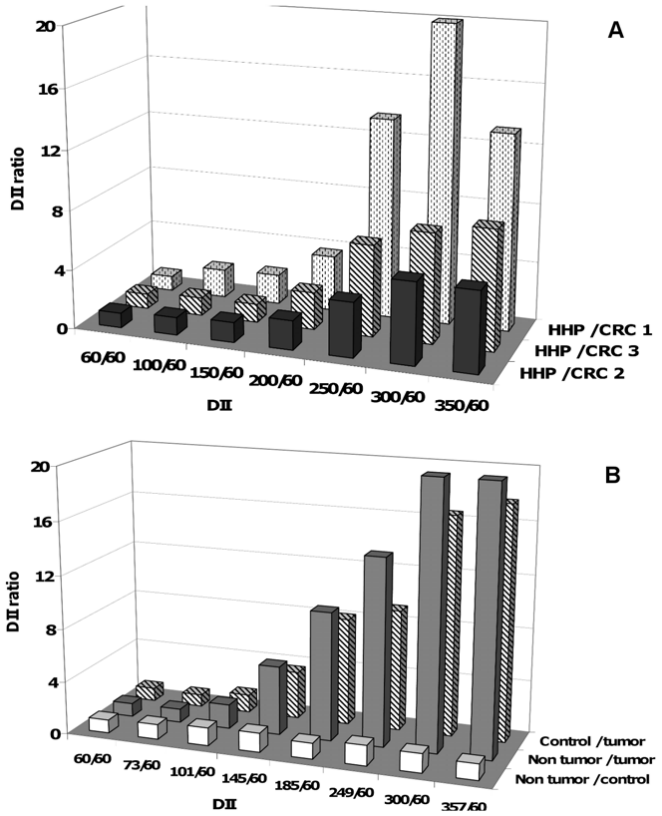
Discrimination between healthy and CRC subjects by comparing DII values. DII ratio between HHP and CRC patients (**A**) and between control non-xenografted and SW620 xenografted mouse plasma (**B**) were determined from DII values represented in Table 1 and S2, respectively. DII of plasma from each CRC patient was compared with the mean of healthy individuals (HHP, n = 9). Mouse DII ratios are determined from the mean of 3 pools from 3 mice (n = 9). The DII was estimated by the ratio of the concentration of amplicons of increasing size and the concentration to the 60 bp amplicons.

**Figure 7:**
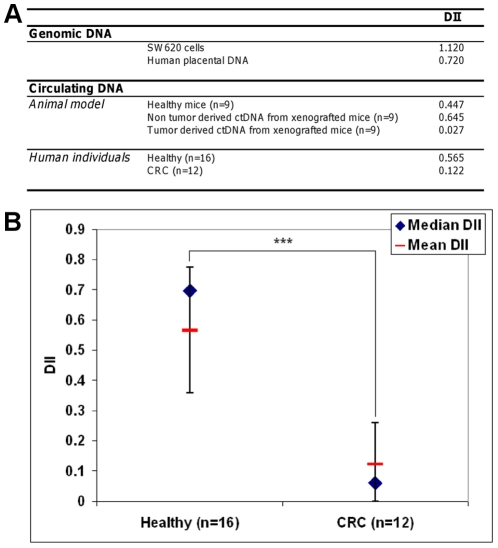
Comparison of the DII values. Comparison of the DII values (**A**) from genomic DNA, and ctDNA from mice plasma (non-xenografted and xenografted) and (**B**) from human plasmas (healthy and CRC). The DII was estimated by the ratio of the concentration obtained by targeting a 300 bp sequence and a 60 bp sequence in a *KRAS* region.

The ratio between the mean HHP DII and the CRC DII was close to 1 for the 60/60 DII and 1.5 for 150/60 DII bp, but sharply increased to 4–19 for the 300/60 DII (Fig. 6). This indicates that the main differences in ctDNA fragmentation between healthy and cancer patients are observed for long amplicons (145–409 and >409 bp). DII from mouse plasmas between the ctDNA concentration of each amplicon >60 bp and that of the 60 bp amplicon was calculated (Table S2). The DII ratios can clearly discriminate tumour ctDNA from non-tumour ctDNA detected in xenografted or non-xenografted mice. The profile of the DII ratios of non-xenografted and xenografted mice remarkably appears similar to those calculated from human healthy individuals and CRC patients. DII appears to similarly discriminate tumour-derived ctDNA from non tumour-derived or control ctDNA. Results strongly confirmed that 300/60 DII seems optimal for discriminating healthy to CRC subjects (Fig. 6). DII mean value of HHP (n = 16) is significatively different from DII mean value of CRC (n = 12) (mean, 0.565 and 0.122, respectively; p<0.001) (Fig. 7). A similar difference was observed in an animal model where mean DII is 0.447 for healthy plasma ctDNA (n = 9), 0.645 for non tumour-derived ctDNA (n = 9) and 0.027 for tumour derived ctDNA (n = 9). The DII values did not seem to be influenced by the genome localization of the amplicons, since comparable results were obtained with amplicons localized in intron 2 and exon 2 of *KRAS* and in exon 15 of *BRAF* (0.036+/−0.006, data not shown).

## Discussion

Here we demonstrate that the length of the amplified fragment dramatically influences the measurement of the ctDNA concentration by Q-PCR. Specifically, a significant higher proportion of tumour ctDNA is detected by primers that target amplicons <100 bp, suggesting that the detection of amplicons <100 bp is more relevant for tumour ctDNA.

Since it was previously established that the shortest ctDNA should theoretically be >180 bp (size of a nucleosome) and since Q-PCR efficiency is conventionally (with genomic DNA) optimal when amplifying sequence of 150–300 bp, most of the previous works relied on the detection of amplicons of about 150 bp when analyzing ctDNA. While Q-PCR assay was often used for ctDNA study, only a couple of reports described the use of primers that amplify segments <100 bp and this choice was based solely on technical aspects linked to primer design and/or to the targeted gene region, but never on the assumption that a higher proportion of <100 bp ctDNA could be present in a specific physiological condition or pathology. Here, we demonstrate, for the first time, the presence of a higher proportion of ctDNA fragments <100 bp that is directly correlated with the increase of ctDNA concentration, particularly in samples from cancer patients. Thus, contrary to the analysis of genomic DNA in which the concentration of quantified DNA is directly proportional to the number of amplified copies, this proportionality varies upon target sequence length in the case of ctDNA from CRC patients. Moreover, the targeting of sequences of 150 to 250 bp (the length commonly chosen for amplification) generates a significant bias by not taking into account up to 80% of the ctDNA amount. Thus, the size profile of ctDNA, as determined by amplifying targets of increasing length, reveals that the optimal detection is obtained with amplicons <100 bp and that a much higher proportion of ctDNA of size ranging from 150 to 400 bp is present in non-tumour ctDNA than in tumour ctDNA. The choice of the size of the amplified DNA region consequently appears critical. Moreover 98% of human CRC tumour ctDNA fragments were <409 bp.

Amplification of fragments of increasing size within the same DNA region showed that non-tumoral ctDNA is less fragmented than tumour ctDNA. In addition, the study revealed that tumour ctDNA fragments have a distinct size profile in comparison to non- tumoral ctDNA and ctDNA from non-xenografted mice. Fragment size profile of non-tumoral, tumoral and control ctDNA are similar in the animal model and in the clinical samples. Specifically, our results suggest that the concentration level and the size distribution profile of ctDNA fragments can be used to discriminate between healthy and cancer patients. Particularly, three ctDNA size groups (<100, 150–400 and >400 bp) might be relevant for such discrimination; exhibiting a higher, much higher and much lower ctDNA amount in cancer patients as compared with healthy individuals, respectively. Thus, the specific analysis of tumour ctDNA can be performed by using a DII calculated from the concentrations of ctDNA of size <100 bp and between 145–409 bp and appears to be optimal when using the 300/60 DII in both xenografted mice and untreated CRC patients. The determination of DII significatively discriminates between HHP and CRC plasmas indicating a higher fragmentation of the corresponding ctDNA. Higher number of subjects is needed to confirm this observation. Ellinger et al determined an integrity index by targeting 124 bp and 271 bp sequences in plasma of prostate [20] and testicular cancer patients [21] and suggested that it witnesses the level of apoptosis among other mechanisms which may release DNA from tumour cells to the circulation. In addition, we think that DII could be very informative in regards to the ctDNA fragment size profile and thus to the observation of the tumour origin.

As a consequence the choice of target length is crucial when determining the DII. This could explain the contradictory results reported by the few previous studies on the clinical use of the integrity index to follow cancer progression that did not allow drawing general conclusions (i.e., estimation of ctDNA fragmentation was found lower [22], [23] equivalent [24], [25] or higher [13], [21], [26], [27] than in controls). As stated by Jung et al [28] those discrepancies might be related to pre-analytical and analytical factors as well as possible bias in the selection of the study groups. We suggest that they may be explained at least partially by the possibility that a large proportion of tumour ctDNA is <100 bp, an option which has never been taken into account. In addition, it is conceivable that our observation might be restricted to the clinical status of the patients whose blood was here examined (stage III mCRC patients not undergoing chemotherapy). Effect of chemotherapy and influence of CRC stage on ctDNA analysis is under investigation in our laboratory.

Our results on the tumour ctDNA size profile disagree with many earlier reports. Most of these works described that ctDNA consisted of fragments of approximatively 100 to 500 bp [29]. To our knowledge, tumour-derived ctDNA lower than 80 bp was previously never examined and quantified on the basis of their higher proportion in ctDNA total population. Precise ctDNA size profiling was reported only in a few works. Liu et al [30], by using microfluidic single molecule spectroscopy, found a size profile for ctDNA fragments >100 bp from stage I and IV lung cancer patient sera that is quite similar to our results. However, they concluded that the greatest discriminatory power occurred at a threshold of 800 bp; no comparison was made with non-neoplastic ctDNA. The authors never focused on ctDNA of size lower than 320 bp, arguing that below 320bp the two curves are similar. D. Lo's team [23] analyzed the size distributions of maternal and fetal DNA in maternal plasma by using a Q-PCR assay with primer sets that amplify sequences from 105 to 798 bp. They suggested that “most of the ctDNA molecules were in the range of 145–201 bp” (which is approximately the nucleosome size). None of the previous reports did examine or discuss the possibility of ctDNA of size lower than 105 bp [23], [24], [26], [29], [30].

Vogelstein's group reported that 1350 to 230 000 ctDNA fragments/ml were found in CRC patients’ plasma samples in comparison to the 1150 to 8280 fragments/ml in disease-free patients [9], confirming the partial overlap between ctDNA level in healthy and CRC individuals described also in other studies [3], [4], [5], [28]. In our study, the fragment number in healthy controls was comparable (2539+/−952 ctDNA fragments/ml, n = 9). Conversely, the ctDNA amount in CRC plasma samples (1,130,920; 298,760; 86,520 and 56,280 fragments/ml as determined by detecting a 60 bp amplicon) was much higher than in control samples, irrespective of the amplicon length. There is a 25-fold disparity when comparing the highest HHP ctDNA value to the lowest CRC ctDNA concentration in our study. This might certainly be due to the Q-PCR analysis that targets a 60 bp sequence and/or the clinical state of the CRC patients group at blood sample time we examined (untreated mCRC). This specificity level may be as well worth further study with a much higher number of clinical samples.

In conclusion, higher and more accurate ctDNA quantification is now made possible enabling a novel examination of ctDNA as cancer biomarker. Size profiling or DII determination could appear as additional potential biomarker, especially during cancer patient monitoring. However, more work has to be done concerning the number of clinical samples and the robustness of those observations to strengthen their relevance for a diagnostic test.

Studies are ongoing in our laboratory to follow these potential biomarkers along with KRAS/BRAF SNP detection in regards to the personalized anti-EGFR therapy for metastatic CRC [31]. This investigation might also be crucial to better understand ctDNA release mechanisms. In addition, this study should be also carried out in other cancer types or in various physiological or pathological conditions that result or lead to ample release of cell-free DNA.

## Supporting Information

Table S1 Characteristics of the selected primers and corresponding amplicons.
(DOC)

Table S2 Values of control DII, non-tumoral DII and tumoral DII in mouse plasma samples determined using the ctDNA concentrations presented in Fig. 3.
(DOC)

Figure S1 CtDNA concentration determined by targeting a 214 bp sequence in intron 2 of mouse *KRAS* and a 189 bp sequence in intron 2 of human *KRAS* in plasma samples from mouse 8 to 16 (previously tested in Fig. 1B).
(DOC)
