# Supplementary material for: High Fragmentation Characterizes Tumour-Derived Circulating DNA
Source: PLoS One. 2011 Sep 6;6(9):e23418. doi: 10.1371/journal.pone.0023418 (PMC3167805; doi:10.1371/journal.pone.0023418)
Supplement: Table S1 — Characteristics of the selected primers and corresponding amplicons. (DOC) [file pone.0023418.s001.doc]

**Table S1: Characteristics of the selected primers and corresponding amplicons.**

| **Species** | **gene** | **location** | **primer name** | **direction** | **sequence 5'-3'** | **Tm (°C)** | **amplicon size (bp)** |
| --- | --- | --- | --- | --- | --- | --- | --- |
| **Human** | ***KRAS*** | intron 2 | KRAS Hf 2 | sense | aatccgtgtgggtcagagag | 59.4 | 189 |
|  |  |  | KRAS Hr 2 | antisense | gaaacaatagccaccctcctt | 57.9 | - |
| **Mouse** | ***KRAS*** | intron 2 | KRAS Mf 3 | sense | ggccaggagtgcattaagac | 59.4 | 214 |
|  |  |  | KRAS Mr 3 | antisense | gcacgtcagatagtctccaaa | 57.9 | - |
| **Human** | ***ACTB*** | intron 8 | HuACTB4353f | sense | GGGACTATTTGGGGGTGTCT | 60.05 | 133 |
|  |  |  | HuACTB4510f | sense | ACCTATGGGATCGTGGCTGT | 61.70 | 290 |
|  |  |  | HuACTB4643r | antisense | CCCCTACCCCAACTTGACTT | 60.22 | - |
|  |  |  |  |  |  |  |  |
| **Human** | ***KRAS*** | Intron 2 | Kras 60 Hf | sense | GCCTGTGACCTACAGTGAAAA | 57.9 | 60 |
|  |  |  | Kras 73 Hf | sense | CCCTACACGTAGAGCCTGTGA | 61.8 | 73 |
|  |  |  | Kras 101 Hf | sense | GAGATGGTGGAAGAACAGGTG | 59.8 | 101 |
|  |  |  | Kras 145 Hf | sense | TGGGCTGTGACATTGCTG | 60.42 | 145 |
|  |  |  | Kras 185 Hf | sense | ATCTGCCTCCGAGTTCCTG | 58.8 | 185 |
|  |  |  | Kras 249 Hf | sense | TGGAAGAGCATAGGAAAGTGC | 57.9 | 249 |
|  |  |  | Kras 300 Hf | sense | GGTCCTTTTCCGTGTGTAGG | 59.45 | 300 |
|  |  |  | Kras 357 Hf | sense | GGCATCTCTAGGACGAAGGT | 59.4 | 357 |
|  |  |  | Kras 409 Hf | sense | GCCTCCCTTTCCAGCACT | 58.2 | 409 |
|  |  |  | Kras 145-300r | antisense | TGACCAAGCAAAACAGACCA | 60.28 | - |
| **Mouse** | ***KRAS*** | Intron 7 | Kras 63 Mr | antisense | GGAGAACAAGCACCCAACAG | 59.4 | 63 |
|  |  |  | Kras 95 Mr | antisense | CAATGCTTCAAACCTCGACA | 55.3 | 95 |
|  |  |  | Kras 150 Mr | antisense | TGCTTCACCTTGTTTGCTCA | 55.4 | 150 |
|  |  |  | Kras 196 Mr | antisense | CCAAGTTCACACTAGAGTCAAAGG | 61.0 | 196 |
|  |  |  | Kras 256 Mr | antisense | CGGGAGTGACAAGAGCATTC | 59.4 | 256 |
|  |  |  | Kras 284 Mr | antisense | GTCAAAGTAATTCCCAACCTCCT | 58.9 | 284 |
|  |  |  | Kras 354 Mr | antisense | GTCATTAAAGTCTACACCTATGTTTCA | 58.9 | 354 |
|  |  |  | Kras 382 Mr | antisense | CCCCGTCTTCAAAGAACATT | 55.3 | 382 |
|  |  |  | Kras 63-382 Mf | sense | AAGAGTGAAGACCCGTGTGC | 59.4 | - |
