# Supplementary material for: High Fragmentation Characterizes Tumour-Derived Circulating DNA
Source: PLoS One. 2011 Sep 6;6(9):e23418. doi: 10.1371/journal.pone.0023418 (PMC3167805; doi:10.1371/journal.pone.0023418)
Supplement: Table S2 — Values of control DII, non-tumoral DII and tumoral DII in mouse plasma samples determined using the ctDNA concentrations presented in Fig. 3. (DOC) [file pone.0023418.s002.doc]

**Table S2:** Values of control DII, non-tumoral DII and tumoral DII in mouse plasma samples determined using the ctDNA concentrations presented in Fig. 3
