# Supplementary material for: High Fragmentation Characterizes Tumour-Derived Circulating DNA
Source: PLoS One. 2011 Sep 6;6(9):e23418. doi: 10.1371/journal.pone.0023418 (PMC3167805; doi:10.1371/journal.pone.0023418)
Supplement: Figure S1 — CtDNA concentration determined by targeting a 214 bp sequence in intron 2 of mouse KRAS and a 189 bp sequence in intron 2 of human KRAS in plasma samples from mouse 8 to 16 (previously tested in Fig. 1B). (DOC) [file pone.0023418.s003.doc]

**Fig. S1:** CtDNA concentration determined by targeting a 214 bp sequence in intron 2 of mouse *KRAS* and a 189 bp sequence in intron 2 of human *KRAS* in plasma samples from mouse 8 to 16 (previously tested in Fig. 1B).
